# Combination of phytochemicals, including ginsenoside and curcumin, shows a synergistic effect on the recovery of radiation-induced toxicity

**DOI:** 10.1371/journal.pone.0293974

**Published:** 2024-01-19

**Authors:** Min-Sung Kim, Su-Jeong Yang, Seo-Yeong Jung, Tae-Yong Lee, Jin-Kyung Park, Yun-Gyeong Park, So-Youn Woo, Seong-Eun Kim, Ryung-Ah Lee

**Affiliations:** 1 Central Research Center, CORESTEMCHEMON Inc., Seoul, South Korea; 2 Department of Microbiology, Ewha Womans University, College of Medicine, Seoul, South Korea; 3 Department of Internal Medicine, Ewha Womans University, College of Medicine, Seoul, South Korea; 4 Department of Surgery, Ewha Womans University, College of Medicine, Seoul, South Korea; University of Michigan Medical School, UNITED STATES

## Abstract

Radiotherapy is commonly used to treat solid cancers located in the pelvis. A considerable number of patients experience proctitis of varying severity, even for a considerable period after radiotherapy. These side effects are often long-lasting or progressively worsen despite multiple therapeutic efforts and are a primary cause of an unexpectedly low quality of life, even after successful cancer treatment. Therefore, this study evaluated the individual and combined efficacy of ginsenoside, curcumin, butyric acid, and sucralfate compounds in treating radiation-induced proctitis. While the candidate compounds did not affect the proliferation and migration of cancer cells, they promoted the recovery of cell activity, including motility. They exhibited anti-inflammatory effects on human dermal fibroblasts or human umbilical vein endothelial cells within in vitro disease models. When each compound was tested, curcumin and ginsenoside were the most effective in cell recovery and promoted the migration of human dermal fibroblasts and cell restoration of human umbilical vein endothelial cells. The combination of ginsenoside and curcumin resulted in cell migration recovery of approximately 54%. In addition, there was a significant improvement in the length of the endothelial tube, with an increase of approximately 25%, suggesting that the ginsenoside-curcumin-containing combination was the most effective against radiation-induced damage. Furthermore, studies evaluating the effects of combined treatments on activated macrophages indicated that the compounds effectively reduced the secretion of inflammatory cytokines, including chemokines, and alleviated radiation-induced inflammation. In conclusion, our study provides valuable insights into using curcumin and ginsenoside as potential compounds for the effective treatment of radiation-induced injuries and highlights the promising therapeutic benefits of combining these two compounds.

## Introduction

Radiotherapy is one of the primary treatments for pelvic malignancies, such as rectal, ovarian, cervical, and bladder cancers [1, 2]. With advances in curative and multimodal treatment options, including radiation treatments, a greater number of patients with these types of cancer live longer than in previous decades [3, 4]. However, 90% of cancer survivors who receive pelvic radiotherapy experience gastrointestinal side effects throughout their lives, with symptoms appearing immediately after radiotherapy and sometimes even after the cancer has been completely cured [5]. The most commonly affected intra-abdominal organs are the distal ileum and lower rectum, resulting in radiation enteritis and radiation proctitis, respectively [6, 7]. Clinical features may include symptoms, such as diarrhea, abdominal pain, bleeding, weight loss, and malnutrition, and may progress to luminal stricture, fistulae, perforation, and uncontrolled bleeding. These side effects can ultimately reduce the quality of life of patients even after successful cancer treatment [8–10].

Various radiotherapy techniques have recently been introduced to minimize radiation sequelae. In radiation therapy, gamma rays are primarily utilized, as they are effective in destroying cancer cells or inhibiting the growth of tumor. These new techniques help reduce the initial sequelae; however, they do not reduce the incidence of chronic radiation therapy induced proctitis, a late complication [11–13]. Although many treatments, including symptom-modifying drugs, anti-inflammatory drugs, and hyperbaric oxygen therapy, have been attempted, more effective recommendations for treating radiation-induced injuries are urgently needed [14, 15].

Recently, the interest in natural compounds as potential therapeutic agents has increased owing to the absence of prominent side effects and unlimited resources [16, 17]. In contrast, chemical manufacturing is relatively complex to develop to avoid safety issues. In this context, we focused on natural plant compounds and explored relevant databases to propose a method to enhance the therapeutic effects of existing drugs by combining them with natural compounds.

As a premise for this combination, we suggested sucralfate based on pre-existing empirical treatment results and clinical trials [18, 19]. Sucralfate is a complex of aluminum and sucrose octasulfate that functions as a mucosal barrier against pepsin and bile acids [20]. Sucralfate protects the mucosa against further injury through physical and cytoprotective effects. Additionally, it protects the proliferative zone and promotes the regeneration of damaged mucosa [21, 22].

Curcumin and ginsenoside are natural compounds derived from *Curcuma longa* and *Panax ginseng*, respectively [23]. Previous studies have reported that curcumin downregulates inflammatory and fibrogenic cytokines and alleviates cutaneous damage in irradiated mice [24]. Moreover, curcumin prevents inflammation and fibrosis in the irradiated lungs of rats [25]. Ginsenosides vary in quantity and type depending on their place of origin, extraction methods, cultivation practices, and growth duration. Different types of ginsenosides exhibit structural differences, resulting in variations in their therapeutic effects. In the case of Rh1 and Rb1, although they both demonstrate excellent anti-inflammatory effects and share structural and functional similarities, they exhibit differences in biological activity. Rb1 mainly focuses on neuroprotection and stress management, while Rh1 is mainly interested in anti-cancer effects and improving brain function. Rh1 is mainly associated with anticancer effects rather than Rb1, and is of interest in studying its anticancer effects. Considering their antioxidant and anti-inflammatory properties, curcumin and ginsenosides are considered noteworthy radiation-protective compounds [26–28]. Considering their antioxidant and anti-inflammatory properties, curcumin and ginsenosides are considered noteworthy radiation-protective compounds.

Butyric acid is the most important metabolite of short chain fatty acids (SCFA). SCFA are the main oxidative fuel of the colonic mucosa and also serve to stimulate colonic mucosal proliferation [29]. Therefore, we combined these compounds to develop an effective therapeutic combination against radiation-induced injuries.

We designed this study to identify chemical compounds that are more effective in reversing radiation-induced intestinal damage from natural compounds like ginsenosides, curcumin, and pharmaceutical compounds such as butyric acid and sucralfate, which are used to destroy cancer cells or inhibit their growth and are also capable of penetrating deep tissues using gamma rays.

## Materials and methods

### Cell culture

Human colon cancer cell lines (HCT116 and SNU-C1), dermal fibroblasts (HDFs), and keratinocytes (HaCaTs) were maintained in Dulbecco’s modified eagle medium-high glucose (Gibco, NY, USA) supplemented with 10% fetal bovine serum (Millipore, MA, USA), 100 units/mL penicillin, and 100 μg/mL streptomycin (Gibco, NY, USA). Human umbilical vein endothelial cells (HUVECs) were cultured in vascular cell basal medium (ATCC, VA, USA) supplemented with endothelial cell growth kit-VEGF (ATCC, VA, USA). Human monocytes (THP-1 cell line) were incubated with Roswell Park Memorial Institute-1640 (Gibco, NY, USA) supplemented with 10% fetal bovine serum (Millipore, MA, USA), 100 units/mL penicillin, and 100 μg/mL streptomycin (Gibco, NY, USA). All cells were maintained at 37°C in 5% CO_2_.

### Irradiation

The radiation irradiation was carried out using the GammaCell 3000 Elan (MDS nordion, Ontario, Canada) machine at a dose rate of 10Gy per 1 minute (laboratory animal center at Hanyang University). After radiation exposure, the cells were placed back in the incubator for 24 hours and then harvested after treatment with trypsin/EDTA (Gibco, NY, USA) for subsequent experiments.

### Cell viability assay

The viability of human colon cancer cells, HCT116 and SNU-C1, was measured using the cell Counting Kit-8 (Dojindo Molecular Technologies, Inc., MD, USA). Cells were seeded in a 60-mm culture dish and irradiated with a single dose of 10 Gy after 24 h. Following 24 h of stabilization, the cells were detached, seeded into a 96-well culture plate, and incubated for 24 h. The cells were then treated with 5, 10, or 50 μmol/L of Rh1, butyric acid, and curcumin (Sigma-Aldrich, MO, USA) for 48 h. Following this, 10 μL of CCK-8 solution were added to wells and incubated for 1–4 h at 37 ˚C. The absorbance was measured at 450 nm using xMark^TM^ (Bio-Rad, Hercules, CA, USA).

### Cell migration assay

The 6.5-mm Transwell inserts with 8.0-μm pores were obtained from Corning (Corning, NY, USA) for the migration assay. The cells were exposed to a single dose of 10 Gy and stabilized for 24 h. The cells were detached and resuspended in 200 μL of opti-MEM (Gibco, NY, USA) supplemented with 10 μmol/L of ginsenosides (Rh1, Rh2, Rb1, or Ro), butyric acid, curcumin, and sucralfate (Sigma-Aldrich, MO, USA) and then seeded in the upper chamber of transwell inserts. The lower chamber of the 24-well plate was filled with a medium containing 10% fetal bovine serum (Millipore, MA, USA) as a chemoattractant. After 24 h of incubation, the cells that migrated to the bottom of the transwell membrane were stained with crystal violet solution, and the stained cells in randomly chosen fields were counted, per group, using an inverted microscope.

### Endothelial cell tube formation assay

The μ-slides for angiogenesis were obtained from Ibidi (Ibidi USA Inc., WI, USA). Ten microliters of Matrigel (Corning, NY, USA) were added to the inner wells of slides and incubated at 37°C for 30 min to 1 h. HUVECs were irradiated with a 10 Gy dose of radiation and were then harvested, and 1 × 10^4^ cells were resuspended in 50 μL media containing 10 μmol/L of Rh1, butyric acid, curcumin, and sucralfate (Sigma-Aldrich, MO, USA). The cells were then seeded on Matrigel-coated slides, and their presence on the gel surface was confirmed. The cells were incubated at 37°C in 5% CO_2_ for 3 h. Thereafter, randomly selected fields in each group were observed under an inverted microscope, and the lengths of the tubes were measured.

### Inflammatory cytokine assay

Human monocytes (THP-1 cell line) were seeded onto a 6-well plate with 3 × 10^5^ cells in 2 mL medium and then stabilized for 24 h. The stabilized cells were differentiated into macrophages by treatment with 10 ng/mL phorbol 12-myristate 13-acetate (Sigma-Aldrich, MO, USA) for 24 h. The macrophages were then exposed to 10 μmol/L of Rh1, butyric acid, curcumin, and sucralfate (Sigma-Aldrich, MO, USA) and incubated for 48 h. The cell supernatant was collected and centrifuged to remove the debris. Inflammatory cytokines, including CXCL10, CCL2, CCL8, IFN-γ-, IL-4, IL-12, and IL-21, were examined using a Bio-plex multiplex system and Bio-plex 200 (Bio-Rad, CA, USA).

### Statistical analysis

Quantitative data are presented as the average ± standard error of the mean (SEM). Statistical analysis for the difference between two groups was conducted using a two sample t-test. For three or more groups, an analysis of variance (ANOVA) was used. All statistical tests were conducted as two-tailed tests. Following the ANOVA analysis, post-hoc analysis was performed using the Tukey method for multiple comparisons. Statistical analyses were performed using GraphPad PRISM Version 5.01 (GraphPad Software Inc., CA, USA). A *p*-value less than 0.05 was considered statistically significant.

## Results

### Effects of ginsenoside, curcumin, and butyric acid on cancer development

**Fig 1:**
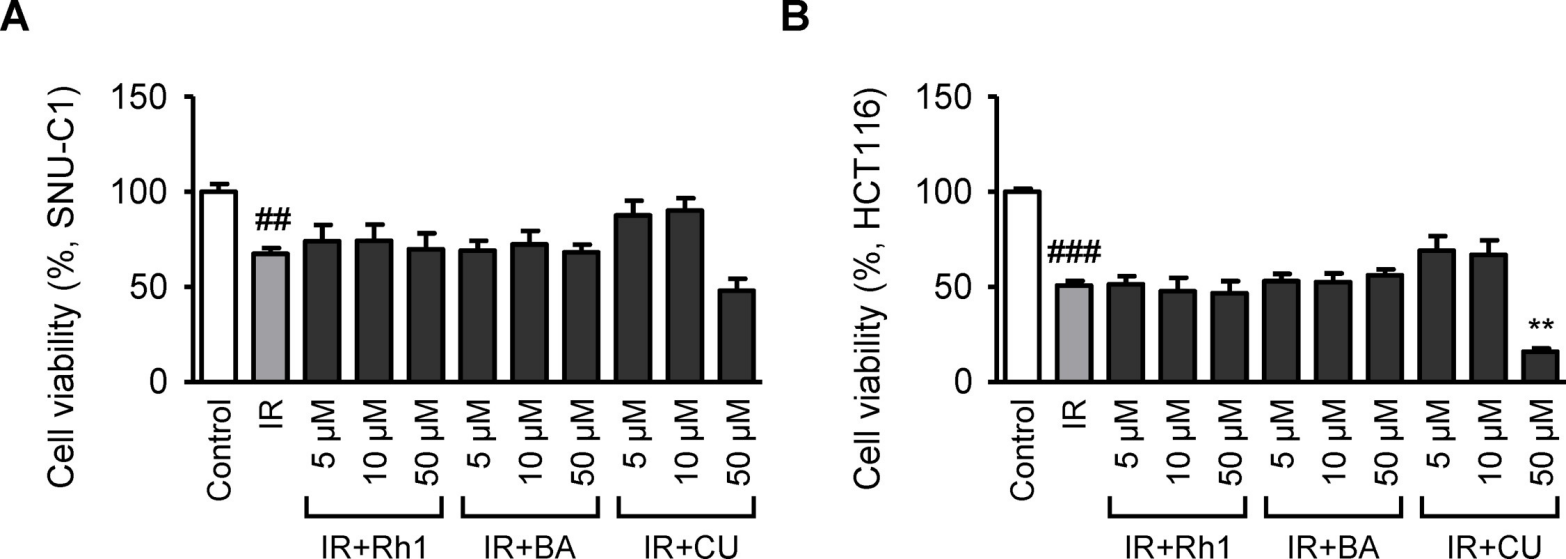
Rh1, butyric acid, and curcumin slightly affect the survival of radiation-exposed colon cancer cells. Cell viability of non-irradiated or irradiated colon cancer cells, (A) SNU-C1 cells, and (B) HCT116 cells, which were treated with 5, 10, and 50 μmol/L of Rh1, butyric acid, and curcumin. Data presented ± SEM. ^##^*p* < 0.01 and ^###^*p* < 0.001 compared to the control group (white columns). ^**^p < 0.01 compared to the IR group (gray columns). Control, non-irradiated; IR, irradiated; BA, butyric acid; CU, curcumin; SEM, standard error of the mean.

**Fig 2:**
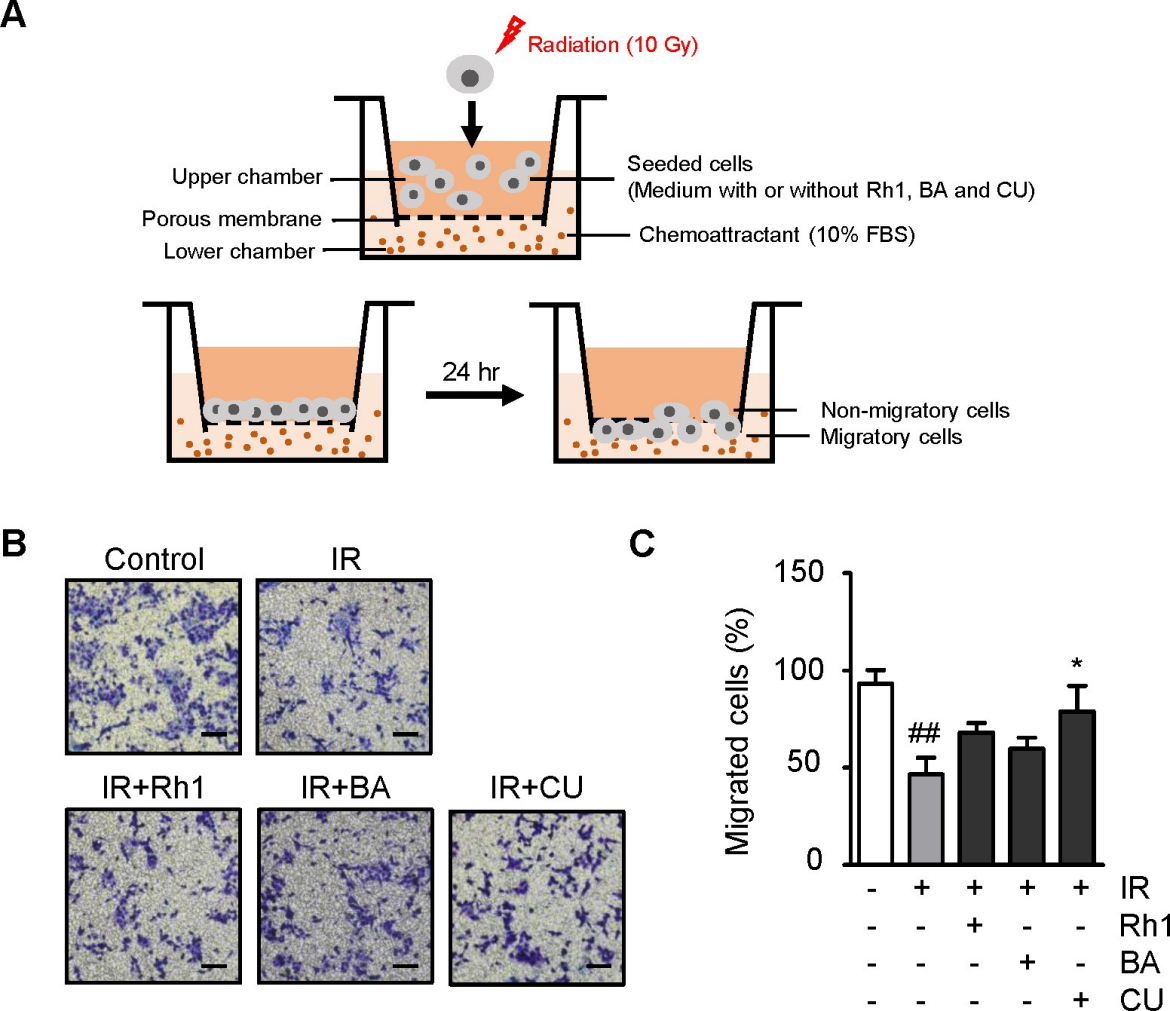
Cancer cell motility after radiation is not restored by Rh1, butyric acid, and curcumin. (A) Schematic presentation of transwell migration assay with irradiated cells. (B) Representative images and (C) graphic diagram of transwell migration of colon cancer cells, SNU-C1 treated with 10 μmol/L of Rh1, butyric acid, and curcumin. Scale bar = 100 μm. Data presented as ± SEM. ^##^*p* < 0.01 compared to the control group (white columns). ^*^p < 0.05 compared to the IR group (gray columns). Control; non-irradiated, IR; irradiated, BA; butyric acid, CU; curcumin SEM, standard error of the mean.

Although radiation is widely used to treat diseases, such as cancer, it can cause severe injuries to normal tissues as a side effect. Therefore, there is a considerable demand for therapeutic compounds that can effectively protect normal tissues from radiation exposure. First, we investigated the effects of the selected natural compounds on cancer cells and confirmed that they did not have any impact on cancer cell development and metastasis. To assess these effects, Rh1, a ginsenoside, butyric acid, and curcumin were used to treat two types of colon cancer cells, and both viability and migration were examined as indicators of cell activity. Radiation exposure decreased the proportion of viable colon cancer cells by approximately 33%(SNU-C1) and 49%(HCT116) (Fig 1A and 1B), (S1 File). Following radiation exposure, 5, 10, or 50 μmol/L of Rh1, butyric acid, or curcumin were applied to colon cancer cells to examine the recovery of the viable cell population. No significant differences were observed in the radiation-alone group (IR), and treatment with 50 μmol/L curcumin significantly reduced cancer cell viability. Cell motility, another marker of cell activity, was investigated using transwell migration analysis (Fig 2A). The colon cancer cells (SNU-C1) that migrated down the membrane were stained and counted (Fig 2B). The results showed that irradiation of colon cancer cells reduced the proportion of migrated cells by approximately 47% (Fig 2C). The group treated with either 10 μmol/L Rh1 or butyric acid did not exhibit a statistically significant difference compared to those treated with IR alone. Conversely, 10 μmol/L curcumin restored approximately 32% of the proportion of migrated cancer cells. However, as curcumin only slightly increased cell motility and did not affect the survival rate, it can be inferred that it did not significantly affect cancer cell activity.

### Restoration from migratory dysfunction by the ginsenoside-curcumin-containing combination

**Fig 3:**
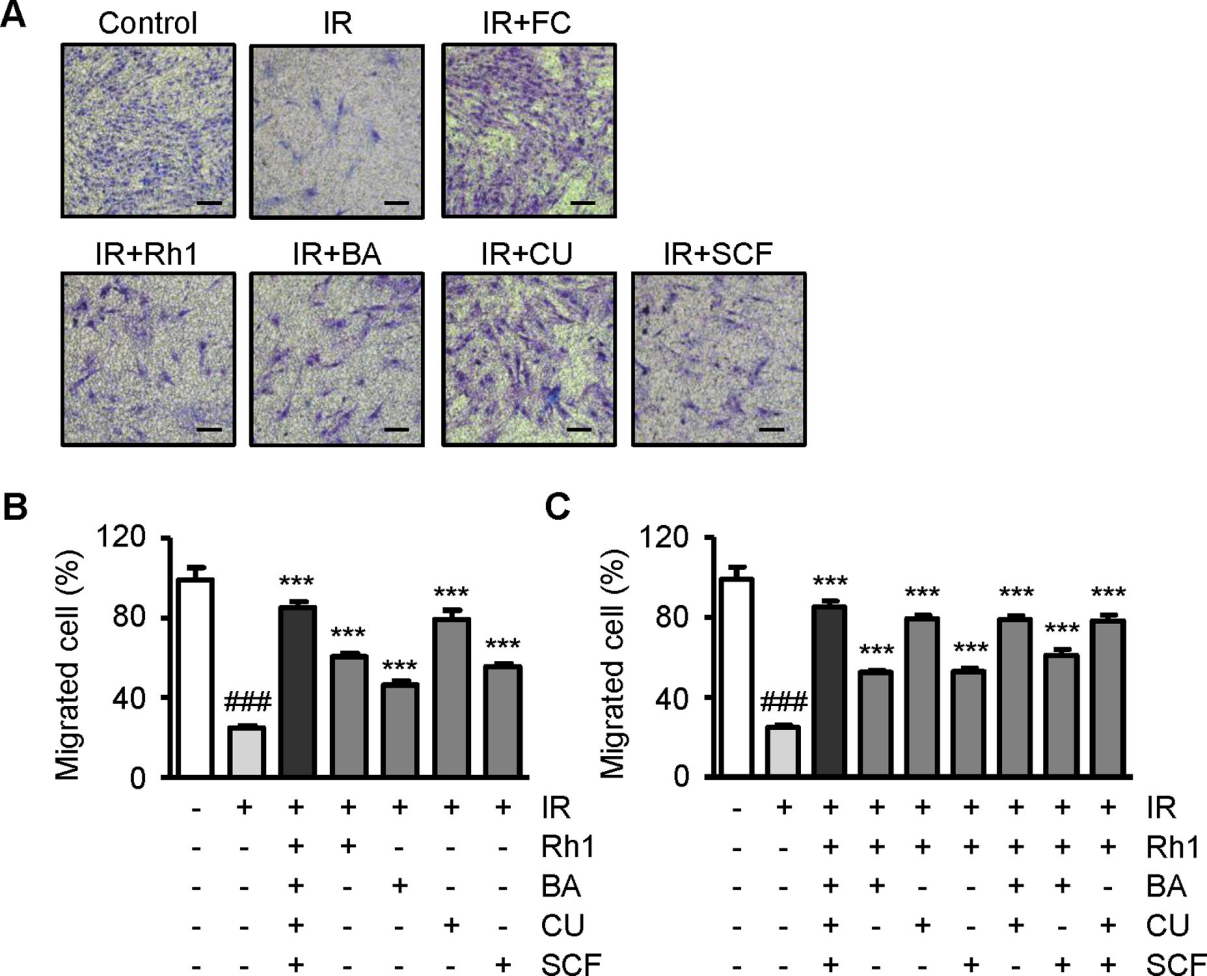
The Full Combination enhances the radiation-damaged cell motility. (A) Representative images and (B) graphic diagram of transwell migration analysis of human dermal fibroblasts (HDFs) treated with Rh1, butyric acid, curcumin, and sucralfate at a concentration of 10 μmol/L. Scale bar = 100 μm. (C) Transwell migration analysis of HDFs treated with various combinations of Rh1, butyric acid, curcumin, and sucralfate. All compounds were treated at a concentration of 10 μmol/L. Data presented as ± SEM. ^###^*p* < 0.001 compared to the control group (white columns). ^***^p < 0.001 compared to the IR group (light gray columns). Control; non-irradiated, IR; irradiated, FC; Full Combination, BA; butyric acid, CU; curcumin, SCF; sucralfate; SEM, standard error of the mean.

We investigated the potential restoration of radiation-induced damage in normal cells (human dermal fibroblasts, HDFs) treated with a combination of Rh1, butyric acid, curcumin, and sucralfate. HDF, which was utilized as a normal cell, is a critical cell type in the composition of rectal tissue, playing a significant role in cell-to-cell boundaries and connections within the tissues. This combination is referred to as the Full Combination, as prior research has suggested the antioxidant or anti-inflammatory effects of these candidates, although their efficacy when used together was not investigated. To evaluate the effects of each compound, HDFs were treated with 10 μmol/L of Rh1, butyric acid, curcumin, and sucralfate, either alone or in combination. Recovery of cell activity, including motility, was assessed using transwell migration analysis (Fig 3A). Exposure of normal cells to 10 Gy of radiation resulted in a reduction of approximately 74% in the proportion of migratory cells. The individual treatment of these cells with 10 μmol/L Rh1, butyric acid, curcumin, or sucralfate, increased the migrated cell ratio by 20–54% compared to the group treated with IR alone. Notably, the order of restoration efficacy was curcumin, Rh1, sucralfate, and butyric acid. The IR-alone group showed 24.8%, while the overall combination group exhibited 85.16%, with a difference of approximately 60% between these two groups (Fig 3B). Subsequently, the cells were treated with combinations of two or three compounds to determine the optimal combination. The combination of Rh1 and curcumin led to a recovery of cell migration of approximately 54%, which was the highest following the Full Combination treatment (Fig 3C).

### Radiation protection by Rh1 among ginsenoside compounds

**Fig 4:**
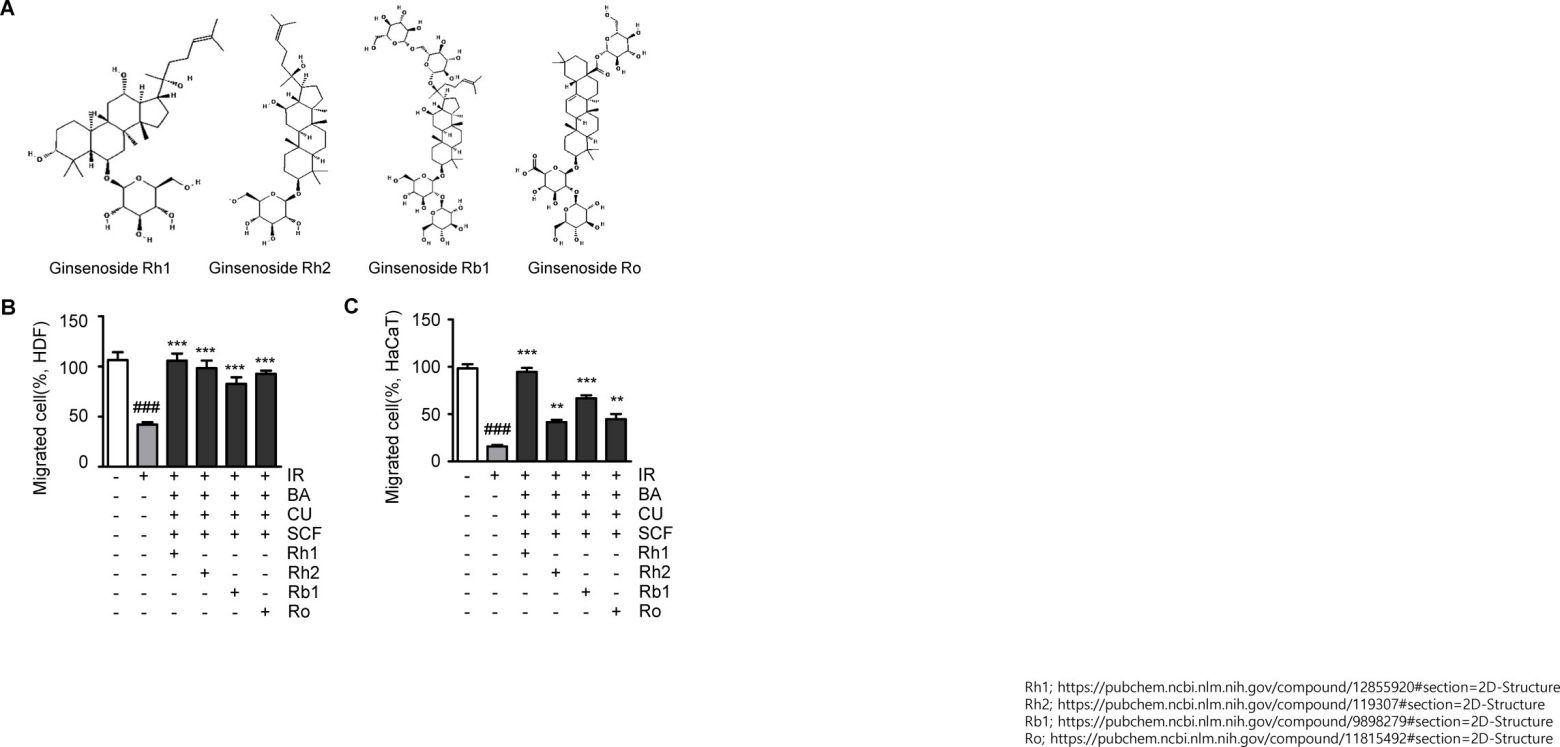
Rh1 is the most effective type of ginsenoside for recovery from radiation-induced damage. (A) Chemical structure of the four types of ginsenosides, Rh1, Rh2, Rb1, and Ro. Two-dimensional structural images from National Center for Biotechnology Information. Pubchem Compound Summary for CID12855920, CID119307, CID9898279, and CID11815492. (B) Transwell migration analysis of (B) human dermal fibroblasts (HDFs) and (C) human keratinocytes (HaCaTs) treated with 10 μmol/L of Rh1, Rh2, Rb1, or Ro in addition to 10 μmol/L of butyric acid, curcumin, and sucralfate. Data presented as ± SEM. ^###^*p* < 0.001 compared to the control group (white columns). ^**^p < 0.01 and ^***^p < 0.001 compared to the IR group (light gray columns). Control; non-irradiated, IR; irradiated, BA; butyric acid, CU; curcumin, SCF; sucralfate; SEM, standard error of the mean.

Ginsenoside is an active ingredient in ginseng, and to date, approximately 17 different ginsenoside compounds have been identified [26]. Accordingly, an investigation was performed to determine whether Rh1 is the most effective ginsenoside for the recovery of radiation-exposed cells by conducting a transwell migration assay, treating the four ginsenosides Rh1, Rh2, Rb1, and Ro with butyric acid, curcumin, and sucralfate (Fig 4A). Radiation reduced the motility of HDFs and the proportion of migrated cells, similar to the results shown in Fig 4 (Fig 4B). When the irradiated cells were treated with various combinations, including Rh1, Rh2, Rb1, or Ro, the ratio of migrated cells was restored by approximately 53% compared to the IR-alone group. In particular, the combination containing Rh1 restored cell motility to a level similar to that of the non-irradiated control group. Another normal cell line, HaCaT, showed increased sensitivity to radiation, with the proportion of migrated cells reduced by approximately 83% (Fig 4C). The combinations with Rh2, Rb1, or Ro restored motility by approximately 26%, 51%, and 29%, respectively. The combination with Rh1 was the most effective in increasing the proportion of migrated cells, approximately 79%, leading to the most significant recovery from radiation-induced motility dysfunction.

### Pivotal roles of ginsenoside and curcumin in cellular recovery after radiation

**Fig 5:**
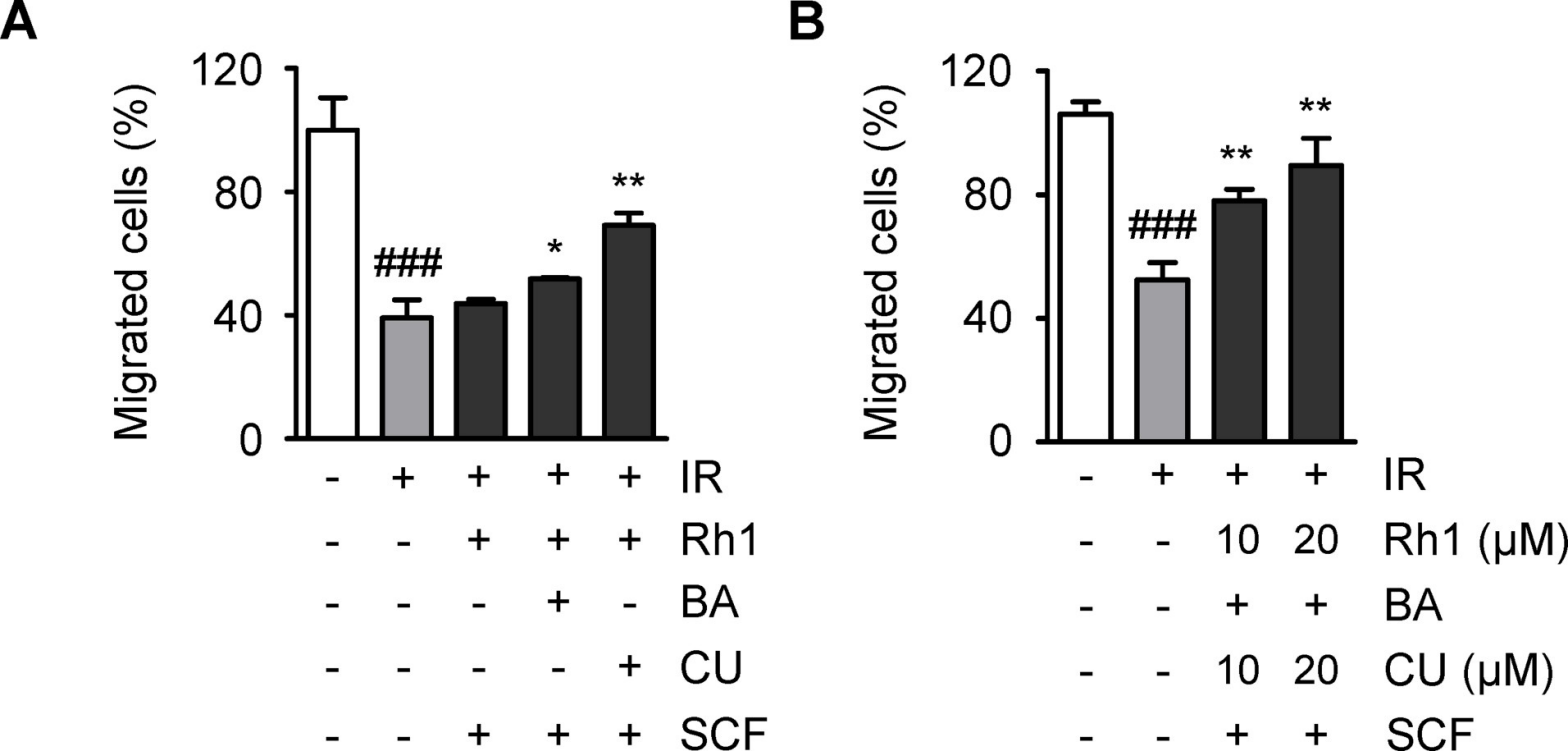
Rh1 and curcumin are the major compounds in restoring cell motility. (A) Cell migration analysis of human dermal fibroblasts (HDFs) treated with different combinations of Rh1, butyric acid, curcumin, and sucralfate at the concentration of 10 μmol/L. (B) Cell migration analysis of HDFs treated with 10 or 20 μmol/L of Rh1 and curcumin along with 10 μmol/L of butyric acid and sucralfate. Data are presented as ± SEM. ^###^*p* < 0.001 compared to the control group (white columns). ^*^p < 0.05 and ^**^p < 0.01 compared to the IR group (gray columns). Control; non-irradiated, IR; irradiated, BA; butyric acid, CU; curcumin, SCF; sucralfate; SEM, standard error of the mean.

To develop more effective therapeutic agents, the most appropriate combination of compounds needed to be determined. To investigate this, the effect of Rh1 and curcumin on the compound combination was analyzed by transwell migration analysis, given the previous results that co-treatment with two or three compounds notably improved radiation-exposed cell motility. The cells were exposed to radiation and treated with Rh1 and sucralfate along with either butyric acid or curcumin, and their motility was observed (Fig 5A). The addition of butyric acid or curcumin resulted in more effective cell recovery, and the combination of Rh1, curcumin, and sucralfate was more restorative on cell motility compared to the combination of Rh1, butyric acid, and sucralfate. These findings suggest that the combined treatment with Rh1 and curcumin provides better efficacy than that with Rh1 and butyric acid. In addition, the recovery of cell migration was found to depend on the concentration of both Rh1 and curcumin, with 10 μmol/L and 20 μmol/L of Rh1 and curcumin restoring the ratio of migrated cells by approximately 26% and 37%, respectively (Fig 5B).

### Endothelial restoration by the ginsenoside-curcumin-containing combination after radiation

**Fig 6:**
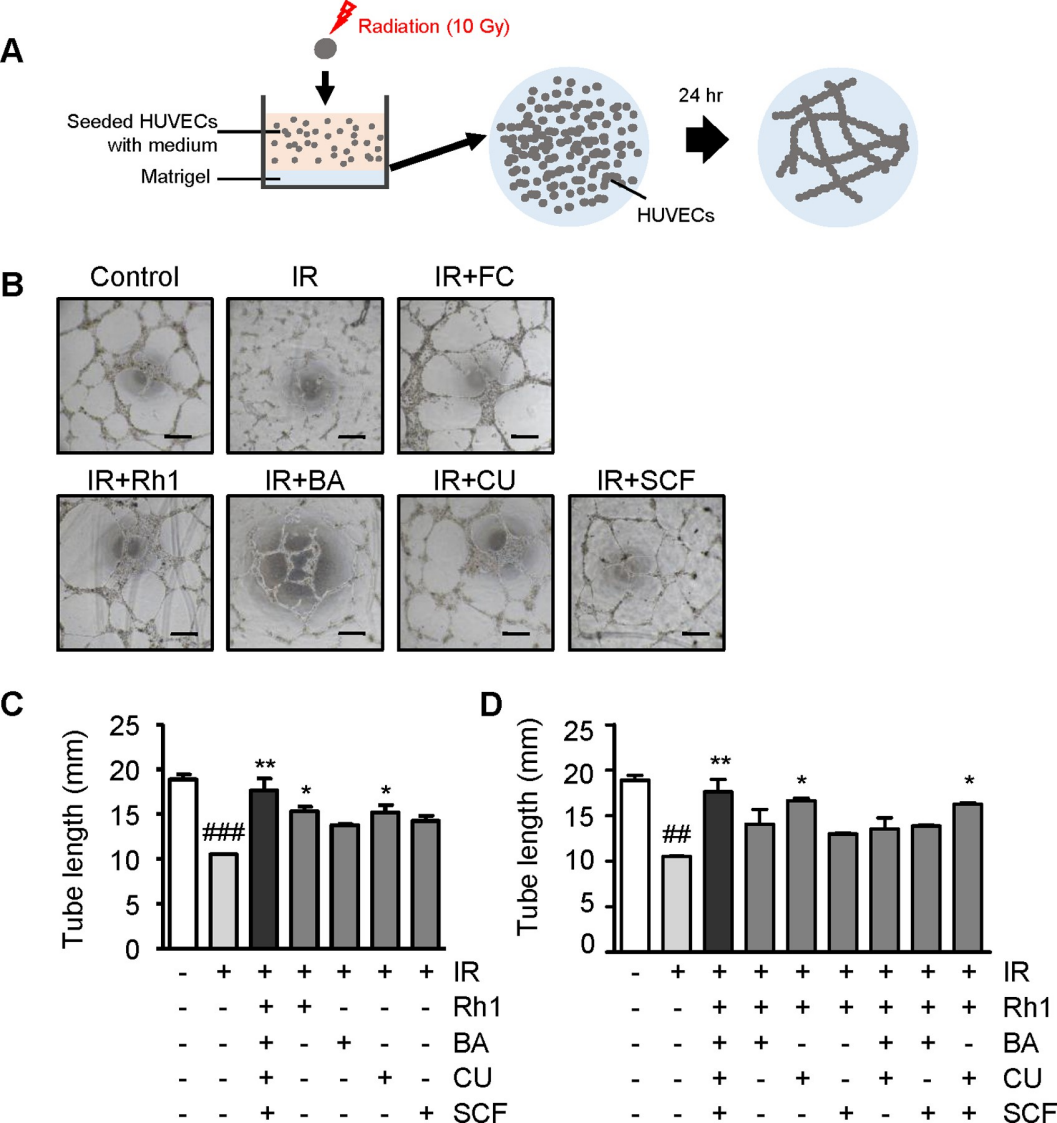
Ginsenoside-curcumin-containing combination improves the irradiated endothelial cell function. (A) Schematic presentation of the tube formation assay in human umbilical vein endothelial cells (HUVECs). (B) Representative images of tube formation in HUVECs. Scale bar = 300 μm. (C) Graphic diagram of tube length in HUVECs. The cells were treated with 10 μmol/L of Rh1, butyric acid, curcumin, and sucralfate. (D) Tube length in HUVECs treated with different combinations of Rh1, butyric acid, curcumin, and sucralfate. All compounds were treated with a concentration of 10 μmol/L. Data are presented as ± SEM. ^##^*p* < 0.01 and ^###^*p* < 0.001 compared to the control group (white columns). ^*^p < 0.05 and ^**^p < 0.01 compared to the IR group (light gray columns). Control; non-irradiated, IR; irradiated, FC; Full Combination, BA; butyric acid, CU; curcumin, SCF; sucralfate; SEM, standard error of the mean.

As an inflammatory response is induced by radiation exposure, alleviating the inflammation is important for treatment. To confirm whether the four candidate compounds had therapeutic effects on radiation-induced inflammation, their effects on the recovery of the endothelium were examined. The endothelium is a radiosensitive tissue and plays a critical role in inflammatory responses, including leukocyte recruitment and cytokine secretion. This implied that radiation-induced damage to endothelial cells may interfere with the recovery response. In this study, we investigated the effects of radiation on tube formation in HUVECs. This was examined using an endothelial cell tube-forming assay. The potential of Rh1, butyric acid, curcumin, and sucralfate to restore endothelial function in radiation-exposed HUVECs was also investigated (Fig 6A and 6B). Treatment with each of the four compounds increased the length of the tubes formed by the radiation-damaged cells (Fig 6C). In particular, the use of Rh1 and curcumin significantly improved the length of the endothelial tube by approximately 25% compared to that of the IR-alone group, which is consistent with previous findings. Furthermore, the Full Combination was more effective in restoring endothelial function than a single compound. Treatment with various combinations of Rh1, butyric acid, curcumin, and sucralfate restored the overall endothelial tube length compared to that in the IR-alone group (Fig 6D). Consistent with previous findings, the combination containing both Rh1 and curcumin restored tube length by 30–32% compared to that in the IR-alone group, while the Full Combination resulted in the most significant restoration of tube length.

### Regulation of inflammatory cytokines by the ginsenoside- curcumin-containing combination

**Fig 7:**
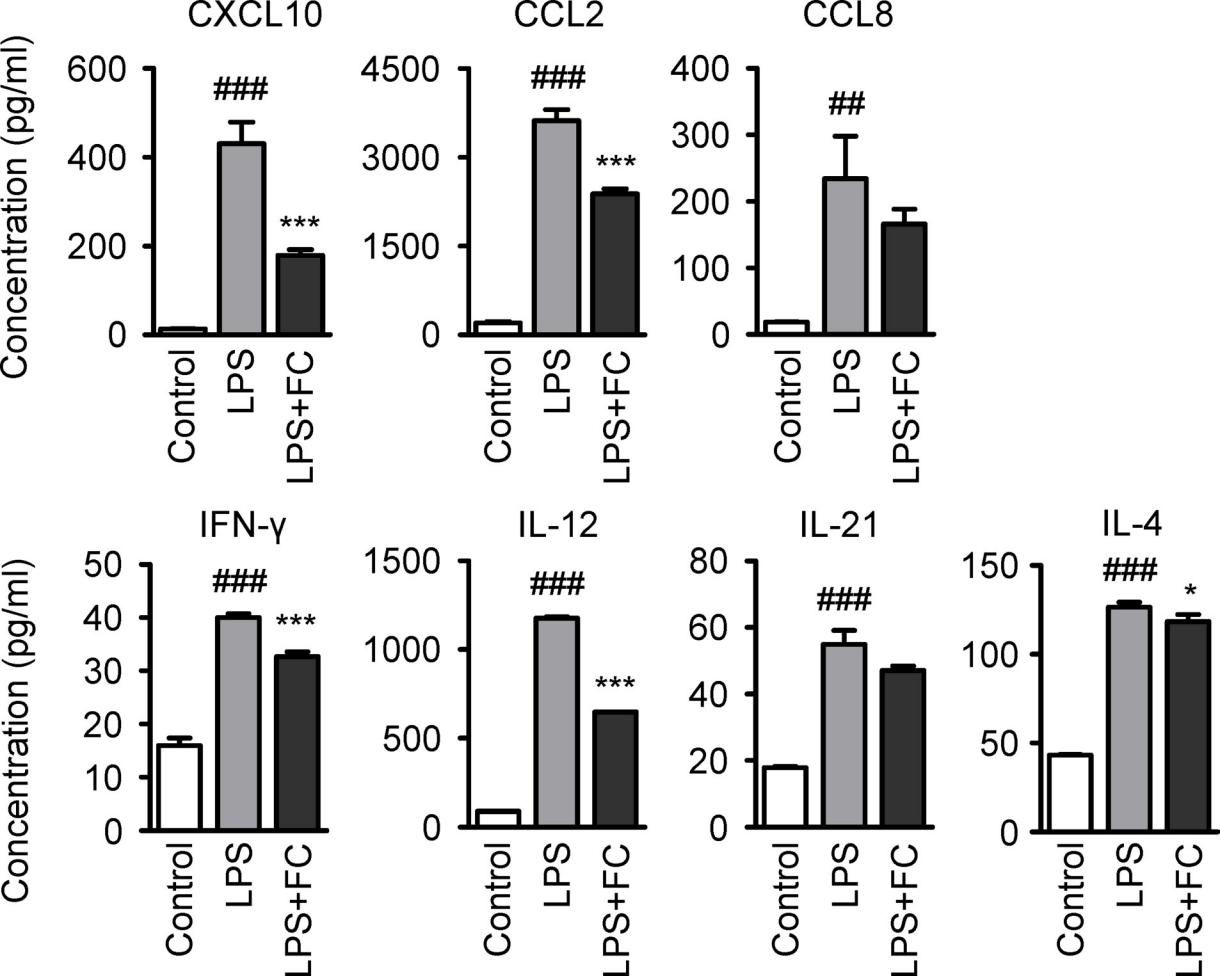
The ginsenoside-curcumin-containing combination reduces macrophage-secreted inflammatory cytokines. Inflammatory chemokine and cytokine analysis of human monocyte (THP-1) derived macrophages treated with lipopolysaccharide (LPS). THP-1-derived macrophages were treated with 10 μg/mL LPS and the Full Combination, all in the concentration of 10 μmol/L. Data are presented as ± SEM. ^##^*p* < 0.01 and ^###^*p* < 0.001 compared to the control group (white columns). ^*^p < 0.05 and ^***^p < 0.001 compared to the IR group (gray columns). Control, untreated LPS, lipopolysaccharide; FC: the Full Combination; SEM, standard error of the mean.

Macrophages play a critical role in regulating intestinal immune homeostasis by manipulating immune responses through the secretion of inflammatory cytokines [28–31]. As macrophage cytokines can promote or suppress tissue inflammation, we investigated cytokine secretion by macrophages following treatment with a combination of ginsenosides and curcumin. The chemokines CXCL10, CCL2, and CCL8, which act as chemoattractants for immune cells, were investigated. Lipopolysaccharide (LPS) treatment of THP-1-derived macrophages increased chemokine secretion by activating the inflammatory response (Fig 7, top row). The Full Combination reduced the concentration of secreted chemokines by approximately 41% compared to that in the LPS-alone group. Inflammation-related cytokines, IFN-γ, IL-12, IL-21, and IL-4, were also observed in the LPS-treated groups alone or with the Full Combination. LPS-induced inflammation increased the expression of these cytokines by at least 2.5 times compared with that of the untreated control group, and treatment with the Full Combination was found to mitigate this increase in expression (Fig 7, bottom row). In particular, the concentrations of IFN-γ and IL-12 decreased by approximately 20% and 45%, respectively, in the Full Combination-treated group. This decrease in secretion was more pronounced than that for IL-21 and IL-4.

## Discussion

Although radiotherapy is commonly used as an anticancer treatment, it can cause severe side effects. Radiation exposure can induce massive cell death in non-cancerous tissues and intensify inflammation [32]. In particular, radiotherapy for gastrointestinal or pelvic cancers can lead to the development of diseases, such as radiation enteritis and radiation proctitis, for which effective therapeutic compounds are currently unavailable. Therefore, we investigated the individual and combined effects of ginsenosides, curcumin, and butyric acid compounds to develop an effective treatment strategy.

To ensure the safety of the candidate compounds, it was necessary to confirm that they did not affect the proliferation or migration of cancer cells. A single treatment with Rh1, butyric acid, or curcumin did not enhance the viability of cancer cells, whereas a high dose of curcumin reduced cell viability. Similarly, the candidate compounds had no significant effect on cancer cell migration except for curcumin, which slightly promoted migration. Consequently, we conclude that ginsenoside, curcumin, and butyric acid could be used as safe therapeutic compounds.

Previous studies have reported the restorative effects of ginsenoside, curcumin, and butyric acid on radiation-induced injuries. Curcumin treatment has been found to treat skin wounds caused by radiation exposure in mice and protect the ileal mucous membrane from radiation in rats [33, 34]. Rd, a type of ginsenoside, has been shown to reduce radiation-induced intestinal epithelial cell apoptosis, and the ginsenosides Rb1, Rb2, Rc, and Rg1, alleviate jejunal injuries after radiation exposure [35, 36]. Additionally, butyric acid, a microbial metabolite, has been evaluated in several clinical trials for its ability to promote recovery from acute radiation-induced diseases [37, 38]. However, studies on the combined efficacy of these compounds are limited. As expected from previous studies, our findings demonstrated that a single treatment with Rh1, butyric acid, curcumin, or sucralfate enhanced the migration of radiation-exposed fibroblasts, and the combined treatment further promoted migration. In addition, the combination containing ginsenoside and curcumin showed similar effects as the Full Combination, regardless of the addition of other compounds, indicating that ginsenoside and curcumin play an important role in the combination effects. Indeed, the restoration of cell migration increased depending on the concentrations of Rh1 and curcumin, confirming that ginsenoside and curcumin are the major components of this radioprotective combination.

The ginsenoside Rh1 is one of the most frequently found components in Korean ginseng [39]. Rh1 has been shown to suppress the inflammatory response by inhibiting inducible nitric oxide synthase and cyclooxygenase-2 [40]. Additionally, Rh1 has been reported to possess antioxidant and anti-inflammatory functions, suggesting that it the most effective in preventing radiation-induced cell damage [41]. Consistently, our results showed that among the four ginsenosides (Rh1, Rh2, Rb1, and Ro), Rh1 demonstrated the best recovery effect in restoring cell migration after radiation exposure.

Given that exacerbation of inflammation is a key pathological mechanism, alleviating the inflammatory response is critical for effectively treating radiation-induced damage [42]. In mice with liver cancer, the combination of curcumin and total ginsenosides is reported to reduce NF-kB-mediated inflammation, indicating its effectiveness in alleviating inflammation [43]. In particular, endothelial destruction by radiation causes leukocyte infiltration and epithelial damage, eventually leading to inflammation [44, 45]. Pravastatin attenuates radiation proctitis by improving endothelial dysfunction [46]. In addition, baicalein extracted from oriental herbal medicines alleviates radiation-induced enteritis by reversing endothelial disorders [47]. This study indicates that combining Rh1 and curcumin most effectively protects endothelial cell tube formation from radiation. The endothelium plays a crucial role in regulating inflammatory responses by controlling leukocyte adhesion and recruitment [48], suggesting that combining ginsenosides and curcumin may exhibit anti-inflammatory effects.

Cytokines are major regulators of inflammatory responses. In the context of radiation proctitis, a known side effect of radiation, the levels of pro-inflammatory cytokines are reportedly elevated in the colon segments of patients compared to those in normal individuals [48]. This study evaluated the effects of combined treatments on activated macrophages. Levels of chemokines, such as CXCL10 and CCL12, and cytokines, including IFN-γ ‐ and IL-12, were further decreased. These findings suggest that the combination treatment may regulate levels of cytokines that attract monocytes or activate effector immune cells and alleviate inflammatory responses.

In summary, our results demonstrated that the ginsenoside-curcumin-containing combination has a restorative effect on radiation toxicity, as suggested by its ability to improve cell survival, migration, and endothelial cell function. Combination treatment also exhibited stronger protective effects against radiation-induced damage. However, these test results have limitations when it comes to explaining in-vivo interactions. Nevertheless, we suggest that the combination of ginsenosides and curcumin, as indicated by test results using various cell types found in the intestine, may hold promise as a therapeutic compound for mitigating radiation-induced damage due to its anti-inflammatory effects.

## Supporting information

S1 File This is the raw data of the all figure results. (XLSX)
